# A Novel Method to Determine the Carbon Isotopic Composition of Inositol Hexaphosphate (Phytate) in Soil by Gas Chromatography–Combustion–Isotope Ratio Mass Spectrometry

**DOI:** 10.1002/rcm.9998

**Published:** 2025-02-09

**Authors:** V. Sarangi, M. Spohn

**Affiliations:** ^1^ Department of Soil and Environment Swedish University of Agricultural Sciences (SLU) Uppsala Sweden

**Keywords:** compound‐specific isotope analysis, inositol hexaphosphate, phytate, soil organic phosphorus

## Abstract

**Rationale:**

Understanding the decomposition of inositol hexaphosphate (phytate), the dominant form of organic phosphorus (OP) in soil, is vital for studying phosphorus (P) cycling in terrestrial ecosystems. However, the lack of multiple stable P isotopes complicates the study of phytate dynamics under natural conditions and over long periods.

**Methods:**

A novel method is presented to determine the carbon isotopic composition of inositol in phytate using compound‐specific isotope analysis. For this purpose, phytate was extracted from soil and purified via ion exchange chromatography, followed by dephosphorylation, derivatization, and analysis using GC‐MS and GC‐C‐IRMS. Pure compounds were also analyzed to assess protocol efficiency, identify isotopic fractionations, and apply isotopic corrections due to derivatization.

**Results:**

Phytate extracted from soil samples was identified using GC‐MS chromatograms. Replicate analyses of the pure compounds indicated that the protocol is highly reproducible. The carbon isotopic composition (δ^13^C) showed a high reproducibility, with values varying by less than 0.5‰ and with no detectable isotopic fractionation during sample preparation. The δ^13^C values of phytate in soil samples reflected the dominant vegetation type (C_3_ or C_4_) growing at the study site.

**Conclusions:**

This study offers a novel approach of determining δ^13^C values of inositol of phytate in environmental samples, offering new opportunities to investigate and quantify OP dynamics based on stable carbon isotopes.

## 1 Introduction

In soil, organic phosphorus (OP) compounds exist in various forms, including orthophosphate esters (e.g., inositol phosphates, nucleotides, sugar phosphates, nucleic acids, phospholipids, phosphoproteins, and glycerophosphates), phosphonates, and orthophosphate anhydrides [1]. Among these, inositol hexaphosphate (phytate) is the dominant form of OP in most soils [2, 3]. Phytate is produced by plants and serves as the primary form of phosphorus (P) storage in their seeds in its free acid form (phytic acid [1, 4]). While phytic acid comprises only a small fraction of OP in plants, it accounts for nearly 50% of the total OP in soils [2, 3]. Therefore, understanding the decomposition, mobility, and bioavailability of phytate in soils is crucial for comprehending P cycling in terrestrial ecosystems [5, 6, 7, 8].

Currently, several spectroscopic (^31^P nuclear magnetic resonance, NMR; inductively coupled plasma atomic–emission spectroscopy, ICP‐AES; and molecular luminescence) and enzyme‐hydrolysis techniques are used to detect and quantify phytate in soil and aqueous samples [9, 10, 11, 12, 13, 14, 15, 16, 17]. To a lesser extent, gas or liquid chromatography–mass spectrometry (GC/LC‐MS) techniques have also been used to study OP at the compound‐specific level [18, 19, 20, 21, 22]. These methods provide insights into the molecular composition of the OP pool or quantifying phytate in natural samples but provide little to no insight into OP dynamics such as decomposition and turnover of phytate in soils. The lack of understanding of OP dynamics in soils is largely due to the lack of multiple stable P isotopes (in contrast to elements such as C or N) and the short half‐lives of P radioisotopes ^32^P and ^33^P (half‐lives of only 14 and 25 days, respectively).

The mineralization of OP, i.e., the conversion of OP into inorganic P, which makes OP available for plant uptake, also called OP decomposition, has traditionally been studied using isotope dilution experiments performed with radioactive P isotopes on incubated soils [23, 24]. While useful, this approach provides only a snapshot of the current OP mineralization under controlled or artificial conditions (e.g., soil oversaturated with water on a shaker). Additionally, the technique has been proven to be very challenging for soils with strong sorption capacity and high microbial activity [23, 25, 26].

In this study, we propose a novel technique for determining the carbon isotopic composition (δ^13^C) of inositol in phytate, which opens new avenues for studying OP dynamics in soils. This approach builds on previous work using carbon isotopes to investigate OP dynamics, such as the decomposition of ^14^C‐labeled OP compounds in soils [27, 28, 29]. While previous studies relied on incubation experiments with highly ^14^C‐enriched compounds added to soil, our method developed a compound‐specific isotope analysis technique to study the natural abundance of carbon isotopes of the dominant OP compound in soils (phytate). This approach compensates for the lack of multiple stable P isotopes by using carbon isotopes to explore OP dynamics in soils. In contrast to incubation studies, which provide only a momentary impression of the P cycle, the stable isotope signature will allow us to study the long‐term dynamics of phytate in soils.

Differences in stable isotope ratios (e.g., ^13^C/^12^C) have been used to quantify the turnover time of various soil organic compounds over long periods and under natural conditions [30, 31, 32, 33, 34]. The ratio between stable isotopes has been used to assess the direction and rate of ecological processes and their effects on the cycling of organic compounds [35, 36]. Here, our approach of determining the carbon isotopic composition of phytate at a compound‐specific level will open new alleys for studying OP dynamics in soils. Specifically, at sites that have experienced vegetation shifts (e.g., from C_3_ to C_4_ plants or vice versa) at a defined point in the past, the natural ^13^C‐labeling technique will enable us to determine the decomposition and turnover of phytate in soils. This approach builds on similar studies that have applied natural ^13^C labeling to quantify long‐term turnover (over periods of 5–80 years) of various soil organic compounds, including lignin [37], carbohydrates [32], *n*‐alkanes [38], plant‐derived fatty acid [39], and soil organic carbon pool [40, 41, 42]. Compared with existing approaches such as isotope dilution or incubation experiments, the natural ^13^C‐labeling technique will provide valuable insights into phytate turnover on a multidecadal scale.

This study aimed to develop a compound‐specific isotopic method to determine the δ^13^C values of inositol in phytate using GC‐C‐IRMS, building on existing analytical methods [19, 21, 22, 43, 44], which goes beyond the quantification of phytate in soil. The specific objectives of the present study include (1) extraction and purification of phytate from soil, (2) quantification and compound‐specific stable carbon isotope analysis of phytate extracted from soil as well as reference compounds using GC‐MS‐IRMS, and (3) determination of correction factors to account for shifts in the isotopic composition during sample preparation and derivatization.

## 2 Materials and Methods

### 2.1 Overview of the Workflow

**FIGURE 1:**
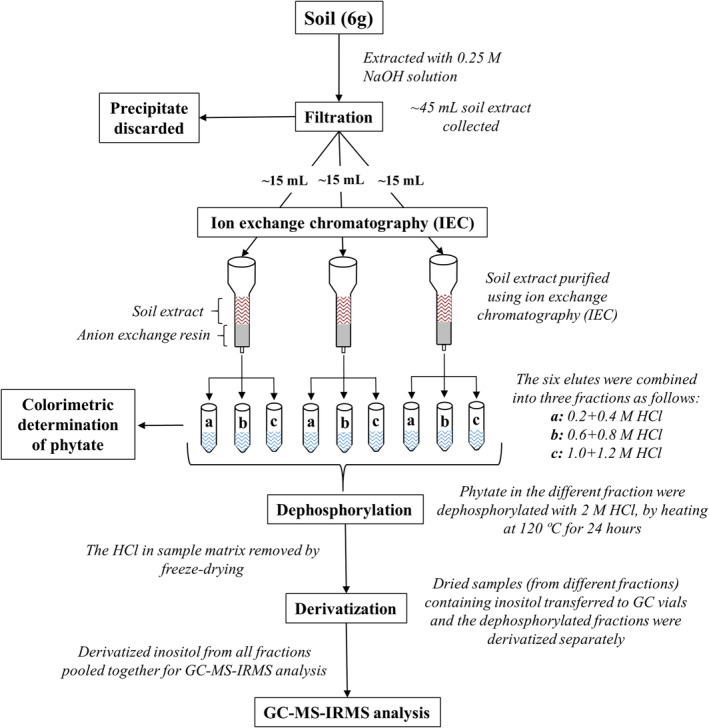
A schematic of the experimental protocol for determining the carbon isotopic composition (δ^13^C) of inositol (from phytate in soil) using a GC‐MS‐IRMS system.

In this study, we developed a method to determine the δ^13^C values of inositol in phytate from soil, while assessing the efficiency at different steps of the protocol. Briefly, the protocol involved extracting phytate, removing the phosphate groups from phytate (dephosphorylation), derivatizing the resulting inositol, and performing compound‐specific analysis using a GC‐MS‐IRMS system (Figure 1). The method closely follows the experimental protocol used by Suzumura and Kamatani [22] to quantify phytate from sediments using GC‐MS.

For soil samples, phytate was extracted using sodium hydroxide (NaOH), with each sample having two replicates (A and B). After extraction, an ion exchange chromatography (IEC) technique was used to purify the soil extracts. Before derivatization, Replicate A was dephosphorylated, while Replicate B was derivatized without prior dephosphorylation. This was done for both the soil samples (Soil 1 and Soil 2). Additionally, three pure compounds were analyzed: two phytates (from rice and maize) and one pure inositol standard. The pure compounds were dissolved in deionized water. For the rice and maize phytates, dephosphorylation was performed before derivatization, while the inositol standard was directly derivatized. Both the pure compounds and soil samples were derivatized using the acetic anhydride method as described by Suzumura and Kamatani [22].

### 2.2 Materials

#### 2.2.1 Soil Samples

We used two soil samples (Soil 1 and Soil 2) with different textures for this study. Soil 1 was collected from a plot at the Lanna experimental station in southwest Sweden and has a fine texture (48% clay) [45]. The field at Lanna has primarily been used to grow C_3_ crops such as winter wheat, peas, spring cereals, and ley [46].

Soil 2 was collected from a cropland in Tänikon, Switzerland. It has a coarser texture (28.7% clay) and is dominated by silver grass (*Miscanthus*), a C_4_ plant [47].

#### 2.2.2 Chemicals

Pure compounds (≥ 98%) of phytic acid sodium salt hydrate (from rice; CAS No.: 14306‐25‐3), inositol‐1,2,3,4,5,6‐hexakisphosphate (from maize; CAS No.: 14306‐25‐3), and inositol (CAS No.: 87‐89‐8; nonanimal source synthetic) were purchased from Merck. Anion exchange resin (AG1‐X8, 100–200 mesh chloride form) and chromatography columns were obtained from Bio‐Rad Laboratories (Richmond, CA, USA). Acetic anhydride (99.5%) and 1‐methylimidazole (≥ 99.0%; GC grade) were purchased from Sigma‐Aldrich. All other chemicals used in this study were of analytical reagent grade.

### 2.3 Extraction and Filtration

Diluted NaOH is commonly used as an extractant in studies of soil OP [48]. Hence, to extract phytate, 6 g of dried and sieved (< 2 mm) soil was suspended in 20 mL of a 0.25 M NaOH solution and shaken in a water bath at 85°C and 140 rpm for 2 h. Afterward, the samples were allowed to cool to room temperature, followed by centrifugation at 8000 *g* for 20 min. The supernatant was decanted and passed through five layers of 23‐μm ashless filter paper. The extraction process for each soil sample was repeated three times. Subsequently, the extract was filtered through syringe filters (0.4‐μm pore size; polytetrafluoroethylene, PTFE, membrane).

### 2.4 IEC

The soil extract was purified using IEC. To prevent clogging of columns, the soil extracts (~45 mL) were divided into three 15‐mL aliquots, and IEC was performed separately for each aliquot (Figure 1). The anion exchange resin (AG1‐X8, 100–200 mesh, chloride form, Bio‐Rad Laboratories) was prepared as a slurry in deionized water and applied to 12‐cm polypropylene chromatography columns with a 20‐mL bed volume and a 10‐mL reservoir [22, 43, 44]. The resin was washed with 10 mL of deionized water. Subsequently, 15‐mL aliquots of the sample extracts were loaded onto the columns. The loaded samples were eluted with 150 mL of deionized water to remove all residual suspended solids and water‐soluble organics. Phytate was eluted from the column using a gradient of HCl (10 mL each of 0.2, 0.4, 0.6, 0.8, 1.0, and 1.2 M HCl) [22, 43, 49]. For convenience and to reduce the number of samples, the six eluates (0.2, 0.4, 0.6, 0.8, 1.0, and 1.2 M HCl) were combined into three fractions (0.2 + 0.4, 0.6 + 0.8, and 1.0 + 1.2 M HCl).

### 2.5 Colorimetric Analysis of Phytate

A rapid colorimetric method based on spectrophotometry (spectral absorbance of the target compound) was used to evaluate the effectiveness of the IEC process (Figure S1). A 660‐μL aliquot of the eluate (collected from IEC) was combined with 330 μL of reagent in a plastic UV‐grade colorimeter cuvette, and the absorbance was measured at 290 nm. The reagent was prepared by dissolving 0.167 g of ferric nitrate nonahydrate in 80 mL of deionized water and adding 20 mL of 1 M perchloric acid [50].

### 2.6 Dephosphorylation

Following IEC, phytate in the extract was dephosphorylated using hydrochloric acid (HCl) according to de Koning [19]. The three eluate fractions (from IEC; see Section 2.4) were dephosphorylated separately. For this purpose, the eluate was transferred to a 50‐mL screw‐cap glass culture tube, and the volume and acid concentration were adjusted to 30 mL and 2 M HCl, respectively [19]. To avoid cracking of glass tubes during dephosphorylation, the loosely capped tubes were preheated in a water bath at 100°C for 25 min. The tubes were then tightly capped and sealed with PTFE tape. The sealed tubes were then placed in an acid‐resistant hot‐air oven at 120°C for 24 h [19]. Thereafter, the HCl was removed from the sample matrix by freeze‐drying the dephosphorylated samples. If the pH of the samples remained below 6 after this step, the samples were redissolved in deionized water and dried under a stream of N_2_ gas at 25°C. The dried and deacidified samples were then redissolved in 1.5 mL of deionized water, transferred to 2‐mL screw‐top GC vials, and evaporated to dryness under a continuous flow of N_2_ gas.

### 2.7 Derivatization of Inositols

The dried samples (both dephosphorylated and non‐dephosphorylated fractions) in the GC vials were suspended in a mixture of deionized water (20 μL) and acetic anhydride (200 μL). Esterification was initiated by adding 20 μL of 1‐methylimidazole and incubating the mixture at room temperature for 15 min. The esterification of inositol (from phytate) to its corresponding acetyl derivative occurred spontaneously. The acetylated samples were then dried under a flow of nitrogen gas at 25°C. The dried samples were redissolved in 200 μL of acetone and transferred to vial inserts for GC‐MS‐IRMS analysis. It should be noted that, for any given soil sample, the dephosphorylated fractions were derivatized separately. The derivatized inositol from all fractions of a given soil sample was subsequently pooled for GC‐MS‐IRMS analysis (Figure 1).

In the following sections, the nonphosphorylated inositol, non‐dephosphorylated phytate, the inositol obtained after the dephosphorylation of phytate, and the derivatized inositol (obtained from dephosphorylated phytate) are referred to as free inositol, phytate, inositol, and derivatized phytate, respectively.

### 2.8 Identification and Quantification of Derivatized Phytate Using GC‐MS

The derivatized phytate was identified and characterized using an ISQ single‐quadrupole mass spectrometer (Thermo Fisher Scientific, Bremen, Germany). The gas chromatograph was equipped with a split/splitless injector and a DB5‐MS UI capillary column (60‐m length, 0.32‐mm i.d., 1‐μm film thickness; Agilent). A total of 1 μL of each derivatized sample was injected in splitless mode using an autosampler (TriPlus RSH) with the inlet temperature set at 280°C. The oven temperature was initially held at 80°C for 5 min, then increased to 320°C at a rate of 6°C min^−1^, and held for 15 min. Helium was used as the carrier gas at a flow rate of 1.2 mL min^−1^, with a total runtime of 60 min. The gas from the GC column was transferred to the MS ion source (at 280°C) via the MS transfer line (at 200°C). Mass spectra were acquired in full‐scan mode (m/z 15–800) using an electron ionization (EI) source operating at 70 eV, with a solvent delay of 5 min and a scan time of 0.2 s per scan.

**FIGURE 2:**
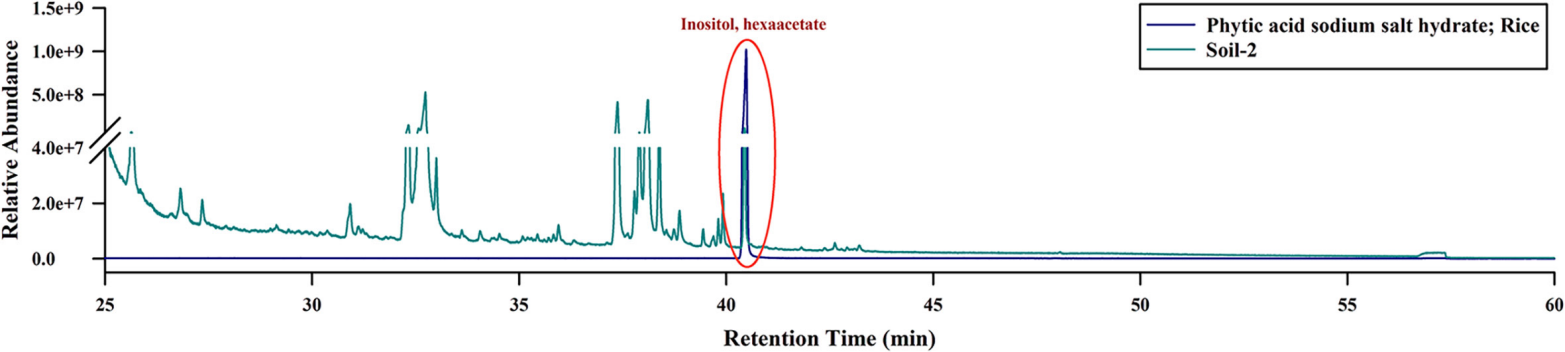
GC‐MS chromatograms from the derivate of inositol (obtained after dephosphorylation and derivatization of phytate) from a pure compound (≥ 98%) of phytic acid sodium salt hydrate (from rice) and a soil sample (Soil 2). Positive identification of the peak in the soil sample was achieved by comparing the retention time (RT) with that of the pure compound.

The peak corresponding to the derivatized phytate in the chromatograms (for soil samples) was identified by comparing its retention time (RT) with that of the pure compound (Figure 2). The identification of the peak was further confirmed by matching the mass spectra of the potential derivatized phytate with spectra from the National Institute of Standards and Technology (NIST) library (NIST 2014), with a probability score of greater than 70%.

The pure compound (inositol) was used to calculate the relative concentrations of phytate in the samples. For calibration, derivatized pure inositol was analyzed at different dilutions (0.4, 0.5, 1.0, 2.0, 4.0, and 6.0 μg μL^−1^), during the analysis of the samples. The peak area of the derivatized inositol was calculated using Thermo Xcalibur Qual Browser (Version 4.0), and a calibration curve of peak area versus concentration was generated (Figure S2). The equation from the regression analysis was then used to calculate the relative concentration of inositol (derived from phytate) in the samples. To determine the concentration of phytate in the samples, a conversion factor based on the molar masses of inositol hexaphosphate and inositol was applied. For the soil samples, the dry weight of the soil used for extraction was factored in to calculate the absolute concentration of phytate in the samples.

### 2.9 Assessing Protocol Efficiency

**FIGURE 3:**
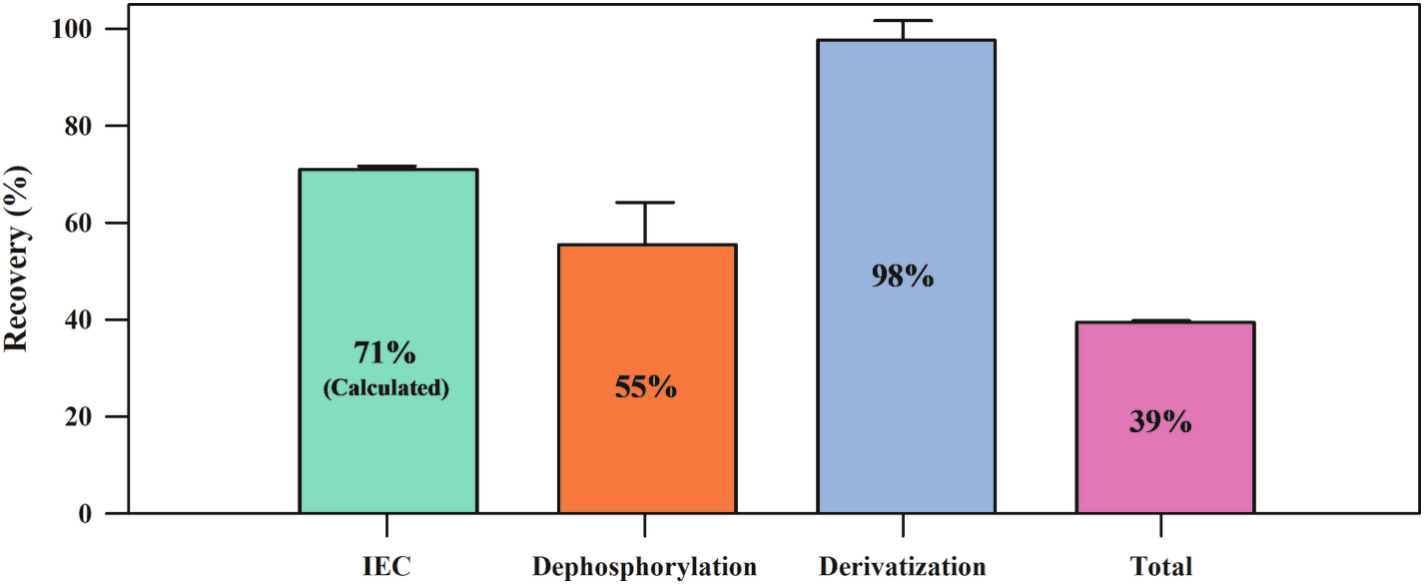
Total and individual recoveries (in percent) at different steps of sample preparation (ion exchange chromatography [IEC], dephosphorylation, and derivatization). Two pure compounds, phytate from rice and inositol, were used to assess protocol efficiency. The error bars indicate the standard deviation (1σ) of the data.

Two pure compounds, phytate from rice and inositol, were used to estimate total as well as individual recoveries during IEC, dephosphorylation, and derivatization. To determine total recovery, a known concentration of pure phytate (from rice) was dissolved in 15 mL of NaOH, and IEC was performed. The fractions collected from the IEC were dephosphorylated, derivatized, and analyzed using GC‐MS, and the concentration of phytate was measured. The theoretical and measured concentrations were compared to calculate the recovery percentage. Recovery during dephosphorylation was estimated by dissolving phytate (from rice) in deionized water to prepare a solution of known concentration, which was then dephosphorylated, derivatized, and analyzed using GC‐MS. The pure compound inositol was used to estimate recovery for the derivatization step. The recovery during purification (IEC) was calculated based on total and individual recoveries. All recovery determinations were performed in replicates, and the mean percentages are reported (Figure 3).

### 2.10 Carbon Isotope Analysis of Derivatized Phytate Using GC‐C‐IRMS

The carbon isotopic composition of derivatized phytate was measured using a Trace GC Ultra instrument coupled to a continuous‐flow isotope ratio mass spectrometer (DeltaV IRMS) via a GC IsoLink (combustion interface) and ConFlo IV interface (all from Thermo Fisher Scientific, Bremen, Germany). An autosampler (TriPlus RSH) was used to inject 1 μL of derivatized sample into the Trace GC Ultra, which was equipped with a splitless injector and a capillary column (DB5‐MS UI). The quantitative and qualitative assessments of derivatized phytate were performed by comparing and matching the mass spectra obtained from GC‐MS and GC‐C‐IRMS, both using the same chromatographic conditions.

The inlet temperature was set at 280°C, and helium was used as the carrier gas with a flow rate of 1.2 mL min^−1^. The GC oven temperature was initially held at 80°C for 5 min, followed by an increase to 320°C at a rate of 6°C min^−1^, where it was held isothermally for 15 min. Derivatized phytate was combusted in the GC IsoLink over nickel, copper, and platinum wires at 1000°C. The CO_2_ produced during the combustion of derivatized phytate was transferred to the DeltaV IRMS for carbon isotope ratio measurement (δ^13^C). A CO_2_ reference gas was mixed into the carrier flow and introduced into the DeltaV IRMS in a series of 13 pulses at the beginning of each analysis to standardize the carbon isotope values. The reference gas was calibrated against an analytical standard (*n*‐decane; Supelco, Bellefonte, PA, USA), which was in turn calibrated with respect to two internationally certified standards (IAEA‐CH‐6 and USGS40). Instrument performance was routinely monitored using the standard (inositol). During the analysis of the derivatized phytate samples, the reproducibility of the δ^13^C values of the standard was found to be ± 0.3‰ (1σ). Samples were analyzed in duplicate to account for any inhomogeneity, and the mean δ^13^C values are reported (Tables 1, 2, and S1). Under the instrument conditions described for GC‐IRMS, we successfully measured reproducible δ^13^C values of derivatized phytate at concentrations ranging from 0.4 to 6.0 μg μL^−1^. Additionally, sample concentrations during isotopic analysis were adjusted by either dilution or concentration under a continuous flow of N_2_ gas.

**TABLE 1:** The δ^13^C values of pure compounds and their derivatives as measured in EA‐IRMS, δ^13^C_(c)_, and GC‐C‐IRMS, δ^13^C_(cd)_, respectively. The δ^13^C values of pure compounds were used to calculate the δ^13^C value of the derivatization reagent, acetic anhydride, δ^13^C_(d)_, using Equation (1).

δ^13^C (‰; VPDB)			
Sample name	Underivatized; EA‐IRMS, δ^13^C_(c)_	Derivatized; GC‐IRMS, δ^13^C_(cd)_	Reagent; calculated, δ^13^C_(d)_
Inositol	−14.0	−45.4 ± 0.3 (*n* = 7)	−61.2 ± 0.4
Phytate (rice)	−30.9 ± 0.1 (*n* = 5)	−51.2 ± 0.5 (*n* = 7)	−61.3 ± 0.6
Phytate (maize)	−24.3 ± 0.1 (*n* = 5)	−49.2 ± 0.2 (*n* = 7)	−61.6 ± 0.2

**TABLE 2:** The δ^13^C values of derivatized phytate in soil samples as determined by GC‐C‐IRMS analysis, δ^13^C_(cd)_. The δ^13^C values of phytate, inositol in phytate, δ^13^C_(c)_, in the soil samples were calculated by substituting the weighted mean of δ^13^C_(d)_ values (calculated from δ^13^C values of pure compounds) and δ^13^C_(cd)_ values (in soil) in Equation (1).

δ^13^C (‰; VPDB)			
Sample name	Derivatized; GC‐IRMS, δ^13^C_(cd)_	Reagent; calculated, weighted mean δ^13^C_(d)_	Derivatized phytate; calculated, δ^13^C_(c)_
Soil 1	−52.1 ± 0.2 (*n* = 3)	−61.5 ± 0.2	−33.3 ± 0.3 (*n* = 3)
Soil 2	−49.4 ± 0.1 (*n* = 2)		−25.2 ± 0.4 (*n* = 2)

Since derivatization alters the isotopic composition of the analyte (inositol in phytate) due to the addition of exogenous carbons during the process, a mass balance equation was applied to correct for isotopic interference from the acetate group (Equation 1) [51, 52]:

(1) $$\delta^{13}C_{\left(c\right)}=\frac{n_{\left(\mathrm{cd}\right)}\delta^{13}C_{\left(\mathrm{cd}\right)}-n_{\left(d\right)}\delta^{13}C_{\left(d\right)}}{n_{\left(c\right)}}$$

where δ^13^C is the carbon isotopic composition of the compounds (in per mille) and *n* is the number of moles of carbon. The subscripts used in the equation are as follows: (c) = compound of interest (inositol in phytate), (cd) = derivatized compound (inositol hexaacetate), and (d) = derivative group (six acetate groups).

For pure compounds, the δ^13^C_(c)_ and δ^13^C_(cd)_ values were determined using EA‐IRMS (see Section 2.10) and GC‐C‐IRMS, respectively. The δ^13^C values obtained were then used to calculate the δ^13^C_(d)_ values using Equation (1). Table 1 presents the calculated δ^13^C_(d)_ values from the replicate analyses of the three pure compounds. For the soil samples, the measured δ^13^C_(cd)_ values (from GC‐C‐IRMS) and the weighted mean of δ^13^C_(d)_ values (calculated from the three pure compounds) were used to correct for isotopic interference from the acetate group and to calculate the δ^13^C_(c)_ values. The standard deviations resulting from the replicate analyses of pure compounds and soil samples were propagated throughout the calculation.

### 2.11 Carbon Isotope Analysis of Pure Compounds Using EA‐IRMS

The bulk carbon isotopic composition of the three pure compounds (inositol and two phytates, from rice and maize) was determined. The pure compounds were packed in tin capsules and combusted in an elemental analyzer (Flash EA 2000, Thermo Fisher Scientific, Bremen, Germany). The carbon isotopic composition was measured by transferring the CO_2_ produced during combustion to a continuous‐flow isotope ratio mass spectrometer (DeltaV, Thermo Fisher Scientific, Bremen, Germany) via a ConFlo IV interface. The δ^13^C values of the pure compounds were measured relative to a reference CO_2_ gas, which was mixed into the carrier flow and introduced into the DeltaV in a series of pulses during each run. The reference gas was calibrated against three internationally certified standards: IAEA‐600, IAEA‐CH‐6, and USGS40. Each sequence of samples was analyzed alongside multiple replicates of two in‐house standards (wheat and maize flour), calibrated relative to IAEA‐600 and IAEA‐CH‐6. These in‐house standards were used to check reproducibility and calibrate the isotope data. During the carbon isotope analysis, a reproducibility of ± 0.15‰ (1σ) was obtained for the in‐house standards. Repeat analyses were performed for the pure compounds, and the details are presented in Table S2.

All the isotopic analyses were conducted in the SLU Stable Isotope Laboratory (SSIL), Sweden. The δ^13^C values of all samples and pure compounds are reported with respect to Vienna Pee‐Dee Belemnite (VPDB).

## 3 Results and Discussion

### 3.1 Extraction and Isolation of Phytate From Soil

We used 0.25 M of NaOH to extract phytate and other OP compounds from the soil samples. Sodium hydroxide is a well‐established solvent for the extraction of OP compounds from soil [53, 54, 55, 56]. Unlike previous methods, which used stronger alkaline solvents (typically 0.5 M of NaOH; for a review, see [48]), we opted for a milder solvent to avoid interference during IEC, where the HCl used to elute phytate could be neutralized by the highly concentrated NaOH. The interaction between NaOH in the matrix and HCl was observed during trial runs of IEC. For example, at higher concentrations, NaOH remained in the IEC column (even after rinsing with 150 mL of deionized water) and reacted with the initial fractions of HCl (0.2 and 0.4 M).

In contrast to previous studies [16, 57, 58], we avoided the addition of ethylenediaminetetraacetic acid disodium salt dihydrate (EDTA) to the extractant because, in pretests with NaOH and EDTA, we observed a large amount of salt precipitates after dephosphorylation, which interfered with the derivatization process. Although NaOH alone may have a lower extraction efficiency than NaOH‐EDTA, this is largely compensated for by repeating the NaOH extraction process three times. In pretests, we found that repeating the extraction step three times increases the amount of extracted phytate.

### 3.2 Dephosphorylation and Derivatization of Phytate for GC‐MS‐IRMS Analysis

We dephosphorylated and derivatized the isolated phytate prior to its analysis using the GC‐MS‐IRMS system. This was necessary because phytate has a very high boiling point due to the six phosphate moieties, making it unsuitable for gas chromatography techniques. To overcome this, the phosphate groups in phytate were removed (dephosphorylation), and the resulting inositol was derivatized to render it volatile for GC analysis.

Phytate samples collected after IEC were dephosphorylated via hydrolysis following the method of de Koning [19]. de Koning [19] demonstrated the effectiveness of hydrochloric acid in dephosphorylating phytic acid by showing that hydrolysis of dimagnesium tetrapotassium phytate with 2 M HCl at 120°C resulted in complete separation of inorganic phosphate from inositol after 24 h. It should be noted that following dephosphorylation, the HCl had to be removed before derivatizing the inositol to ensure complete and effective derivatization [22]. We achieved this by freeze‐drying the dephosphorylated samples. If the pH of the samples remained ≤ 6 after this process, the samples were redissolved in deionized water and dried under a stream of N_2_ gas at 25°C. Although it is possible to remove HCl by boiling the extract to dryness at 100°C and then adding deionized water, we found that the inositol was susceptible to thermal degradation at such high temperatures and extremely low‐pH conditions.

To increase the volatility of inositol for reliable GC‐MS‐IRMS analysis, we derivatized the dephosphorylated phytate (inositol). In the present study, we have used the acetic anhydride method to derivatize the inositol [22]. We used this method due to its proven applicability for GC‐MS analysis of phytate extracted from soil [21, 22]. The acetic anhydride method successfully converted inositol (from phytate) into inositol hexaacetate, making it suitable for gas chromatography analysis.

Inositol naturally occurs in both free and phosphorylated forms (as phytate). Since inositol forms upon dephosphorylation of phytate, the presence of free inositol in soil samples could potentially complicate the quantification and determination of δ^13^C values using GC‐MS‐IRMS. To address this, we prepared two replicates for each soil sample: One replicate was derivatized after dephosphorylation, while the other was derivatized without prior dephosphorylation. This approach ensured reliable quantification of inositol in the soil samples. The analysis confirmed that no free inositol was detected in the soils by GC‐MS‐IRMS (more details in Section 3.3), consistent with previous findings that free inositol is generally absent in agricultural soils [32, 59]. The absence of free inositol in the soil samples could also be linked to the extraction method employed in this study. For instance, previous studies have utilized H_2_SO_4_ (an acidic solvent) to isolate sugars (chemically similar to inositol) such as mannose, galactose, and glucose from various soil fractions and plant organs [59]. In contrast, we used an alkaline solvent (0.25 M NaOH), which may have been ineffective at extracting free inositol. It is also possible that free inositol, if present, was extracted in minor amounts but decomposed during the extraction procedure or was washed away during IEC, along with residual suspended solids and water‐soluble organics (see Section 2.4). Regardless, the absence of free inositol in soil samples simplifies the interpretation of carbon isotope values, making the analysis more straightforward.

### 3.3 Analysis of Inositol From Phytate With GC‐MS: Identification, Reproducibility, Quantification, and Recovery

**FIGURE 4:**
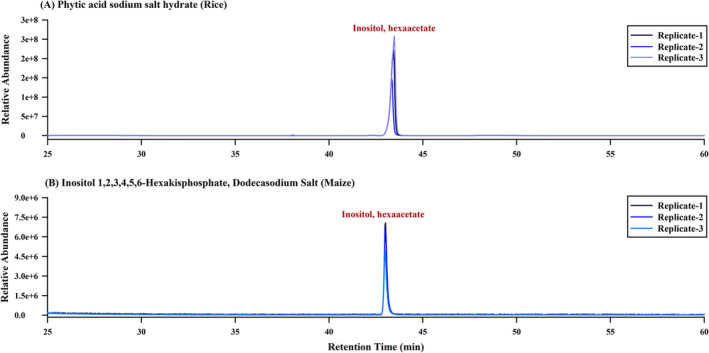
GC‐MS chromatograms of derivate of inositol from (A) rice and (B) maize. Inositol was obtained by dephosphorylation of phytate and derivatized using the acetic anhydride method. Replicate analyses demonstrate the reproducibility of the protocol.

**FIGURE 5:**
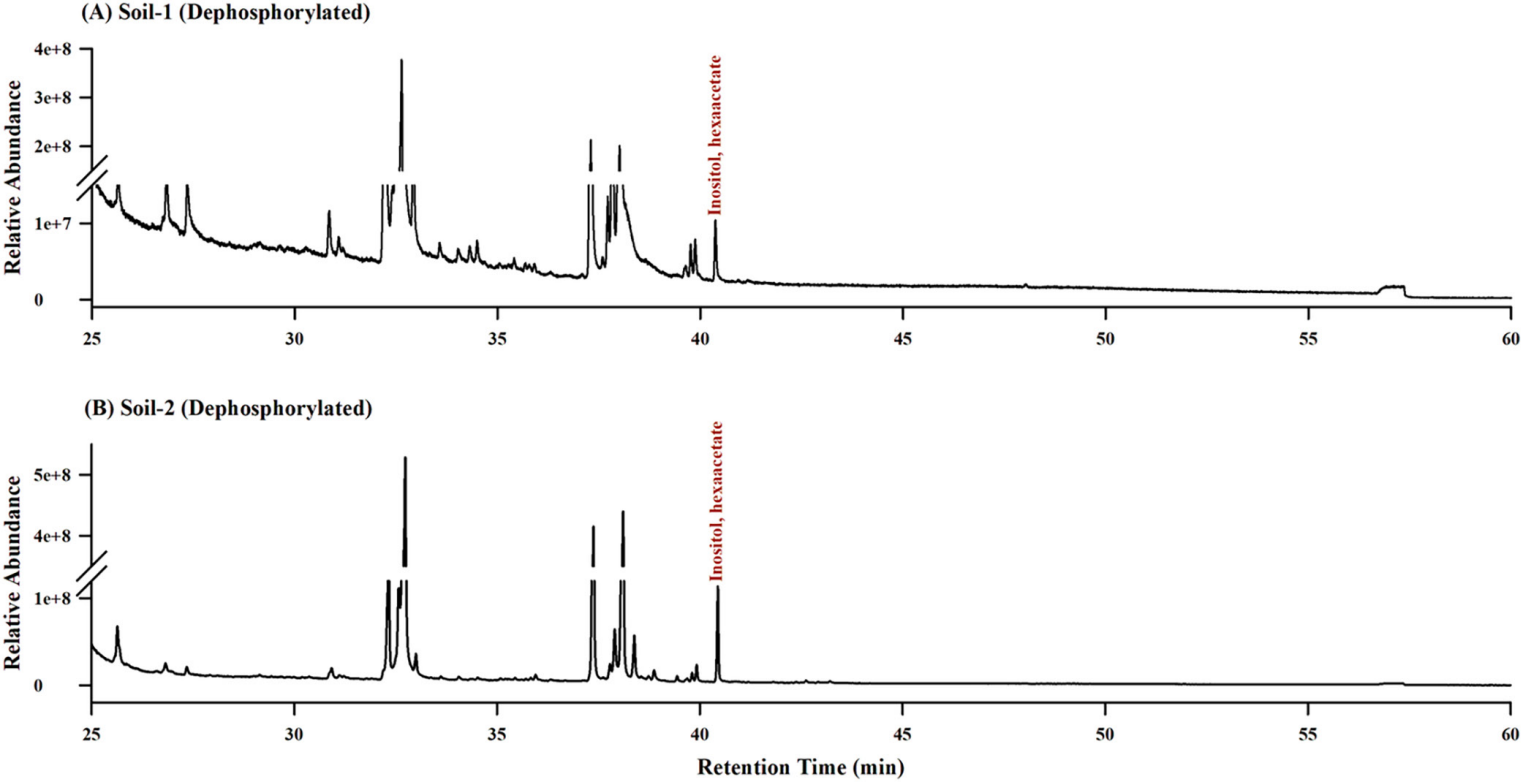
GC‐MS chromatograms of extracts from (A) Soil 1 and (B) Soil 2, showing the relative abundance of derivatized phytate in the soil samples.

Typical GC‐MS chromatograms obtained from the analysis of inositol hexaacetate derived from pure compounds (phytic acid sodium salt hydrates from rice and inositol‐1,2,3,4,5,6‐hexakisphosphate from maize) and soil samples are shown in Figures 4 and 5. The chromatograms exhibit a well‐defined peak for derivatized phytate without interference from other peaks, demonstrating the high resolution achieved through gas chromatography using the proposed method. The GC‐MS method effectively resolved the inositol peak from other organic compounds with similar chemical compositions, such as glucose and fructose (Figure S3). Moreover, no peaks corresponding to derivatized phytate were observed in the GC‐MS chromatogram for the non‐dephosphorylated sample, confirming the absence of free inositol in the soil samples (Figure S3). Figure 4 presents representative GC‐MS chromatograms obtained from the repeated analysis (*n* = 3) of inositol hexaacetate from the pure compounds. The consistent results across replicates demonstrate the reproducibility of the developed method.

To better understand the isolation and elution of phytate during IEC, eluates collected from different acid fractions were analyzed separately. The GC‐MS chromatograms of phytate (derived from rice) eluted at various HCl concentrations during IEC showed that nearly all the phytate was eluted with 0.6 and 0.8 M HCl (Figure S4). Similar findings were reported by Suzumura and Kamatani [22], who observed that most phytate and its stereoisomers (*myo*‐, *chiro*‐, and *scyllo*‐inositol phosphates) were preferentially eluted at higher acid strengths (> 0.5 M of HCl) during IEC of extracts from marine sediments.

We observed a disparity in the elution pattern of phytate between the pure compound (rice‐derived phytate) and soil samples during IEC (Figures S1, S3, and S4). In soil samples, a major proportion of phytate was eluted at lower acid strengths of 0.2 and 0.4 M HCl (Figure S3). This elution pattern in soil samples was further corroborated by results from colorimetric analysis (Figure S1). These observations contrast with previous studies, where lower acid strengths (≤ 0.5 M HCl) preferentially eluted a mixture of inorganic P (e.g., KH_2_PO_4_) and inositol phosphates with fewer phosphate moieties (inositol tetraphosphate or pentaphosphate) [22, 49]. However, GC‐MS analysis of soil extracts confirmed the presence of phytate in the 0.2 and 0.4 M of acid fractions (Figure S3).

We hypothesize that the observed differences in phytate elution between pure compounds and soil extracts are due to impurities in the soil extracts. For example, Suzumura and Kamatani [22] used hypobromite oxidation during the extraction of phytate, which oxidized and removed coexisting impurities. They also used a coprecipitation procedure with CaCl_2_, further purifying the phosphate compounds after extraction. In contrast, the absence of these steps in our protocol likely allowed complex interactions between phytate and other organic compounds in the soil extracts, which influenced its elution pattern during IEC. Yet the effects of these impurities seem to be rather small since they affect only the RT but not the efficiency of the IEC. Furthermore, the elution pattern of phytate during IEC does not impact our findings, as all eluted fractions were combined prior to isotopic analysis (see Section 2.7).

The pure compounds (phytate from rice and inositol) were used to assess the efficiency of the developed protocol. Individual recoveries of 71 ± 0.7% (purification), 55.5 ± 8.7% (dephosphorylation), and 97.7 ± 4.0% (derivatization) were estimated at various sample preparation steps (Figure 3). While the measured phytate represents only a fraction of the extracted phytate, the method allows us to determine the carbon isotope ratio of the compound, which is the focus of the method presented here. The concentration of phytate was found to be higher in Soil 2 (64.3 ± 0.2 μg g^−1^; 1σ; *n* = 2) than in Soil 1 (58.3 ± 0.5 μg g^−1^; 1σ; *n* = 3).

### 3.4 Measurement of δ^13^C Values of Inositol From Phytate Using GC‐C‐IRMS: Evaluating Applicability and Determining Correction Factor

**FIGURE 6:**
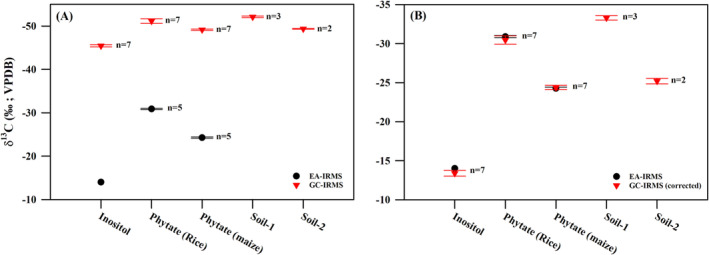
(A) The δ^13^C values of pure compounds (measured in EA‐IRMS) and the carbon isotopic composition of derivatized phytate in pure compound and soil samples determined using GC‐C‐IRMS. (B) The corrected δ^13^C values of inositol (in phytate) from pure compounds and soil samples. The measured and calculated δ^13^C values in pure compounds indicate no additional isotopic fractionation during sample preparation. The symbols represent the mean values, and the error bars indicate the standard deviation (1σ) of the data.

The bulk δ^13^C values of pure compounds, inositol, and phytate (underivatized) from rice and maize were measured by EA‐IRMS to be −14.0‰, −30.9 ± 0.1‰ (1σ; *n* = 5), and −24.3 ± 0.1‰ (1σ; *n* = 5), respectively (Table 1 and Figure 6A). The δ^13^C values of phytate differed based on the photosynthetic pathways (C_3_ or C_4_), highlighting its potential for identifying the source of OP in soil. However, derivatization of inositol (from phytate) is known to alter its original δ^13^C value [60, 61]. For example, the carbon isotopic compositions of derivatized inositol, pure inositol, and phytate from rice and maize were measured to be −45.4 ± 0.3‰ (1σ; *n* = 7), −51.2 ± 0.5‰ (1σ; *n* = 7), and −49.2 ± 0.2‰ (1σ; *n* = 7), respectively (Figure 6A). Thus, correcting for changes in δ^13^C values due to the introduction of new carbon‐bearing functional groups during derivatization is essential. The δ^13^C values of inositol derivatives are derived from the carbon isotopic compositions of the inositol as well as the contributing carbon from the derivatizing reagents (acetic anhydride). Therefore, a generalized stoichiometric mass balance equation (Equation 1) can be used to calculate the δ^13^C values of inositol (from phytate) using the δ^13^C values of both the derivatized compound and the derivatizing reagents [52].

The derivatization process is often associated with isotopic fractionation due to the kinetic isotope effect (KIE [52]). The KIE causes a fractionation of carbon isotopes in the derivatized compound and must be accounted for in reliable isotopic measurements [52, 60, 61, 62]. Our finding that the calculated (using Equation 1) and experimentally obtained δ^13^C values of pure compounds were similar suggests little to no isotope fractionation during the derivatization reaction (Figure 6B). Since KIE is primarily associated with a nonquantitative reaction of the derivatizing reagent, varying the amount of analyte could influence the magnitude of KIE [63]. To test this, we measured the carbon isotopic composition of derivatized pure inositol at different dilutions (ranging from 0.4 to 6.0 μg μL^−1^). The derivatization of the inositol replicates was performed using identical reagent volumes and reaction conditions. The δ^13^C value of derivatized inositol across the different concentrations (0.4, 0.5, 1.0, 2.0, 4.0, and 6.0 μg μL^−1^) was measured at −45.5 ± 0.2‰ (1σ; *n* = 6). Since the precision of the GC‐C‐IRMS was determined to be ± 0.3‰ (1σ), it can be concluded that KIE, if present, does not significantly affect the δ^13^C value of derivatized phytate when the analyte concentration ranges between 0.4 and 6.0 μg μL^−1^.

In the soil samples, the δ^13^C values of derivatized phytate were measured to be −52.1 ± 0.2‰ (1σ; *n* = 3) and −49.4 ± 0.1‰ (1σ; *n* = 2) for Soils 1 and 2, respectively (Table 2 and Figure 6A). As the relative concentration of inositol (from phytate) in the soil samples ranged between 0.47 and 0.53 μg μL^−1^, KIE would not cause significant variation in their carbon isotopic compositions. Therefore, Equation (1) was applied to calculate the δ^13^C value of inositol (from phytate). For Soils 1 and 2, the δ^13^C values of inositol were calculated to be −33.3 ± 0.3‰ (1σ; *n* = 3) and −25.2 ± 0.4‰ (1σ; *n* = 2), respectively (Table 2 and Figure 6B).

Apart from isotopic fractionation, the presence of stereoisomeric forms of inositol hexaphosphate can also influence the interpretation of δ^13^C values of derivatized phytate. In soil, inositol hexaphosphate predominantly occurs in four stereoisomeric forms: *myo*‐, *scyllo*‐, *neo*‐, and D‐*chiro*‐isomers [17]. Previous studies have shown that *myo*‐inositol hexaphosphate is the most abundant isomer, accounting for up to 90% of the total inositol hexaphosphate in soil [17, 64, 65]. The general abundance of these isomers follows the order *myo* > *scyllo* > D‐*chiro* > *neo*, with the latter three isomers collectively representing 10%–50% of the total inositol hexaphosphate in soil [55].

In this study, however, we measured the carbon isotopic composition at a compound‐specific level using GC‐IRMS, which is capable of resolving the peaks of various stereoisomeric forms of inositol hexaphosphate. Similar observations were reported by Suzumura and Kamatani [22], who identified separate GC peaks with different RTs for *myo*‐, D‐*chiro*‐, and *scyllo*‐inositol hexaphosphates in marine sediments. Moreover, during the analysis of soil samples, we used a pure inositol compound (specifically *myo*‐inositol) to identify the peak corresponding to *myo*‐inositol hexaphosphate (derivatized phytate) in the chromatograms. Additionally, when identifying the peak using the NIST library, the mass spectrum of the target peak matched that of *myo*‐inositol hexaacetate with a probability score exceeding 70%. Based on this, we used the carbon isotopic composition of the *myo*‐inositol hexaacetate peak in the soil samples for our interpretation.

The δ^13^C values of inositol (from phytate) in the soil samples showed good correspondence with the photosynthetic pathways of the dominant vegetation at the sampling sites (see Section 2.2.1). This indicates that the δ^13^C values of phytate in the soil samples can be reliably used to identify the source of OP. While we refer to phytate, i.e., inositol hexaphosphate, here, it should be noted that the method quantifies also the isotopic signature of other inositol phosphates with fewer phosphate moieties that are less abundant in soil [66].

### 3.5 Implication and Potential Applications

In this study, we developed a novel methodology for determining the carbon isotopic composition of phytate in soil. The protocol can also be applied to other natural samples, such as sediments or plants, making it useful for investigating the origin and dynamics of phytate in various ecosystems, including aquatic environments.

The δ^13^C values of phytate can be used to trace its origin in soils at sites where the vegetation changed from C_3_ to C_4_ plants or vice versa. In addition, at sites where such a change occurred only once and at a specific point in the past, the δ^13^C value of a compound can even be used to calculate its turnover time in the soil [30, 31, 32, 35]. This approach relies on the inherent difference in δ^13^C values between the biomass of plants that use the C_3_ or C_4_ photosynthetic pathways. Generally, C_3_ plants are more depleted in ^13^C compared with C_4_ plants [31, 35, 67]. For example, the δ^13^C value of phytate from rice (a C_3_ plant) is 6.6‰ lower than that of maize, which follows the C_4_ photosynthetic pathway (Table 1). Since the δ^13^C values of phytate differ between C_3_ and C_4_ plants, the natural ^13^C‐labeling technique can be used to trace its origin and determine its turnover time in soils at sites where vegetation changed from C_3_ to C_4_ plants or vice versa. For instance, if a cropland previously cultivated with C_3_ crops undergoes a vegetation change to C_4_ crops, a general mass balance calculation can be applied. By analyzing the temporal changes in the δ^13^C value of a specific compound (such as phytate) in the soil and using the end‐member δ^13^C values of the compound from C_3_ and C_4_ plants, it is possible to track decomposition‐driven loss of C_3_‐derived compounds over time and determine their turnover time [37, 68]. Thus, this technique opens up new opportunities to test hypotheses about element cycling in soils. For example, it can be used to examine the hypothesis that carbon covalently bound to phosphorus has a longer transit time in soil organic matter than carbon in phosphorus‐free organic compounds [69]. This can be achieved by determining the turnover of phytate in soils and comparing it with the turnover time of bulk organic carbon.

The method developed here can also aid in identifying the sources of OP in aquatic systems through stable isotope fingerprinting. In water bodies with high external P loading, the mineralization of sediment‐hosted OP can lead to long‐term eutrophication [18, 70]. However, due to limitations in current analytical methods, the contribution of sediment‐hosted phytate to inorganic P is not well understood. By determining the δ^13^C values of phytate in sediments and potential sources, we can now more accurately identify natural and anthropogenic sources of OP and quantify their contributions to aquatic ecosystems.

## 4 Conclusions

We proposed a novel GC‐C‐IRMS–based method to determine the carbon isotopic composition of phytate in soil. The developed methodology relies on the extraction, purification, dephosphorylation, and derivatization of phytate. The derivatization process was devoid of any KIE as indicated by the similarity in the measured and calculated δ^13^C values of phytate from pure compounds. Therefore, the developed method is suitable for the isotopic analysis of phytate in soil and other natural samples.

## Author Contributions

**V. Sarangi:** conceptualization, methodology, data curation, investigation, validation, visualization, writing – original draft, writing – review and editing, formal analysis, project administration, software. **M. Spohn:** conceptualization, methodology, supervision, funding acquisition, project administration, resources, writing – review and editing, visualization, data curation.

## Supporting information


**Table S1** The δ^13^C values of derivatized phytate in pure compounds and soil samples. The table shows the isotope values for replicate analyses. NA = not analyzed.
**Table S2** The δ^13^C values of pure compounds (in replicates) in bulk form measured using EA‐IRMS.
**Figure S1** Absorbance values (Abs.) for phytate eluted from the ion exchange column with increasing concentrations of HCl from (A) rice and (B) soil sample (Soil 1). The absorbance values for eluates collected from different acid fractions indicate that nearly all of the phytate from the pure compound was eluted with 0.6 and 0.8 M HCl, whereas in the soil sample, phytate was primarily eluted at the initial phase with 0.2 and 0.4 M HCl.
**Figure S2** A calibration curve obtained using the peak area and relative concentration of the pure inositol compound. The equation from the regression analysis was then used to calculate the relative concentration of inositol (derived from phytate) in the samples.
**Figure S3** The GC‐MS chromatogram of extracts of (A, B) Soil 1 and (C, D) Soil 2. Samples were obtained as eluates from ion exchange chromatography at different HCl concentrations. Two eluates were each pooled as indicated in the figure legend. The eluates were derivatized using the acetic anhydride method by Suzumura and Kamatani [61]. The chromatograms show the relative abundance of various organic phosphorus compounds (in derivatized form) in non‐dephosphorylated (A, C) and dephosphorylated samples (B, D).
**Figure S4** The GC‐MS chromatogram of the derivate of inositol from rice (obtained after dephosphorylation and derivatization of phytate) collected as eluate from ion exchange chromatography at different acid strengths (with NaOH as a matrix). Eluates collected from different acid fractions were pooled as indicated in the legend. The chromatograms show that almost all of the phytate was eluted with 0.6 and 0.8 M HCl.

## Data Availability

The data that support the findings of this study are available in the Supporting Information of this article.
