# Supplementary material for: A Novel Method to Determine the Carbon Isotopic Composition of Inositol Hexaphosphate (Phytate) in Soil by Gas Chromatography–Combustion–Isotope Ratio Mass Spectrometry
Source: Rapid Commun Mass Spectrom. 2025 Feb 9;39(9):e9998. doi: 10.1002/rcm.9998 (PMC11808226; doi:10.1002/rcm.9998)
Supplement: Supplementary file 1 — Table S1 The δ13C values of derivatized phytate in pure compounds and soil samples. The table shows the isotope values for replicate analyses. NA = not analyzed. Table S2 The δ13C values of pure compounds (in replicates) in bulk form measured using EA‐IRMS. Figure S1 Absorbance values (Abs.) for phytate eluted from the ion exchange column with increasing concentrations of HCl from (A) rice and (B) soil sample (Soil 1). The absorbance values for eluates collected from different acid fractions indicate that nearly all of the phytate from the pure compound was eluted with 0.6 and 0.8 M HCl, whereas in the soil sample, phytate was primarily eluted at the initial phase with 0.2 and 0.4 M HCl. Figure S2 A calibration curve obtained using the peak area and relative concentration of the pure inositol compound. The equation from the regression analysis was then used to calculate the relative concentration of inositol (derived from phytate) in the samples. Figure S3 The GC‐MS chromatogram of extracts of (A, B) Soil 1 and (C, D) Soil 2. Samples were obtained as eluates from ion exchange chromatography at different HCl concentrations. Two eluates were each pooled as indicated in the figure legend. The eluates were derivatized using the acetic anhydride method by Suzumura and Kamatani [61]. The chromatograms show the relative abundance of various organic phosphorus compounds (in derivatized form) in non‐dephosphorylated (A, C) and dephosphorylated samples (B, D). Figure S4 The GC‐MS chromatogram of the derivate of inositol from rice (obtained after dephosphorylation and derivatization of phytate) collected as eluate from ion exchange chromatography at different acid strengths (with NaOH as a matrix). Eluates collected from different acid fractions were pooled as indicated in the legend. The chromatograms show that almost all of the phytate was eluted with 0.6 and 0.8 M HCl. [file RCM-39-e9998-s001.docx]

**Table-S1:** The δ^13^C values of derivatized-phytate in pure compounds and soil samples. The table shows the isotope values for replicate analyses. N.A.= Not analyzed.

| **δ^13^C (‰; VPDB); Derivatized; GC-IRMS** | | |
| --- | --- | --- |
| **Sample Name** | **δ^13^C_(cd)_** | **δ^13^C_(cd)_ (Repeat)** |
| **Inositol** | −45.8 | N.A. |
|  | −45.1 | N.A. |
|  | −45.4 | N.A. |
|  | −45.6 | N.A. |
|  | −45.6 | N.A. |
|  | −45.1 | N.A. |
|  | −45.7 | N.A. |
| **Phytate (Rice)** | −51.4 | -51.5 |
|  | −50.7 | -50.6 |
|  | −51.1 | -51.3 |
|  | −50.1 | -51.5 |
|  | −51.7 | -52.0 |
|  | −51.2 | -51.6 |
|  | −50.8 | -50.8 |
| **Phytate (Maize)** | −48.8 | N.A. |
|  | −49.0 | N.A. |
|  | −49.1 | -49.3 |
|  | −49.1 | -49.2 |
|  | −49.3 | -49.0 |
|  | −49.5 | -49.1 |
|  | −49.2 | -49.1 |
| **Soil-1** | −52.0 | -52.1 |
|  | −52.0 | -51.8 |
|  | −52.3 | -52.4 |
| **Soil-2** | −49.5 | -49.3 |
|  | −49.3 | -49.3 |

**Table-S2:** The δ^13^C values of pure compounds (in replicates) in bulk form measured using EA-IRMS

| **δ^13^C (‰; VPDB); Underivatized; EA-IRMS** | |
| --- | --- |
| **Sample Name** | **δ^13^C_(c)_** |
| **Inositol** | −14.0 |
| **Phytate (Rice)** | −30.8 |
|  | −30.8 |
|  | −30.9 |
|  | −31.0 |
|  | −31.1 |
| **Phytate (Maize)** | −24.1 |
|  | −24.2 |
|  | −24.4 |
|  | −24.4 |
|  | −24.4 |


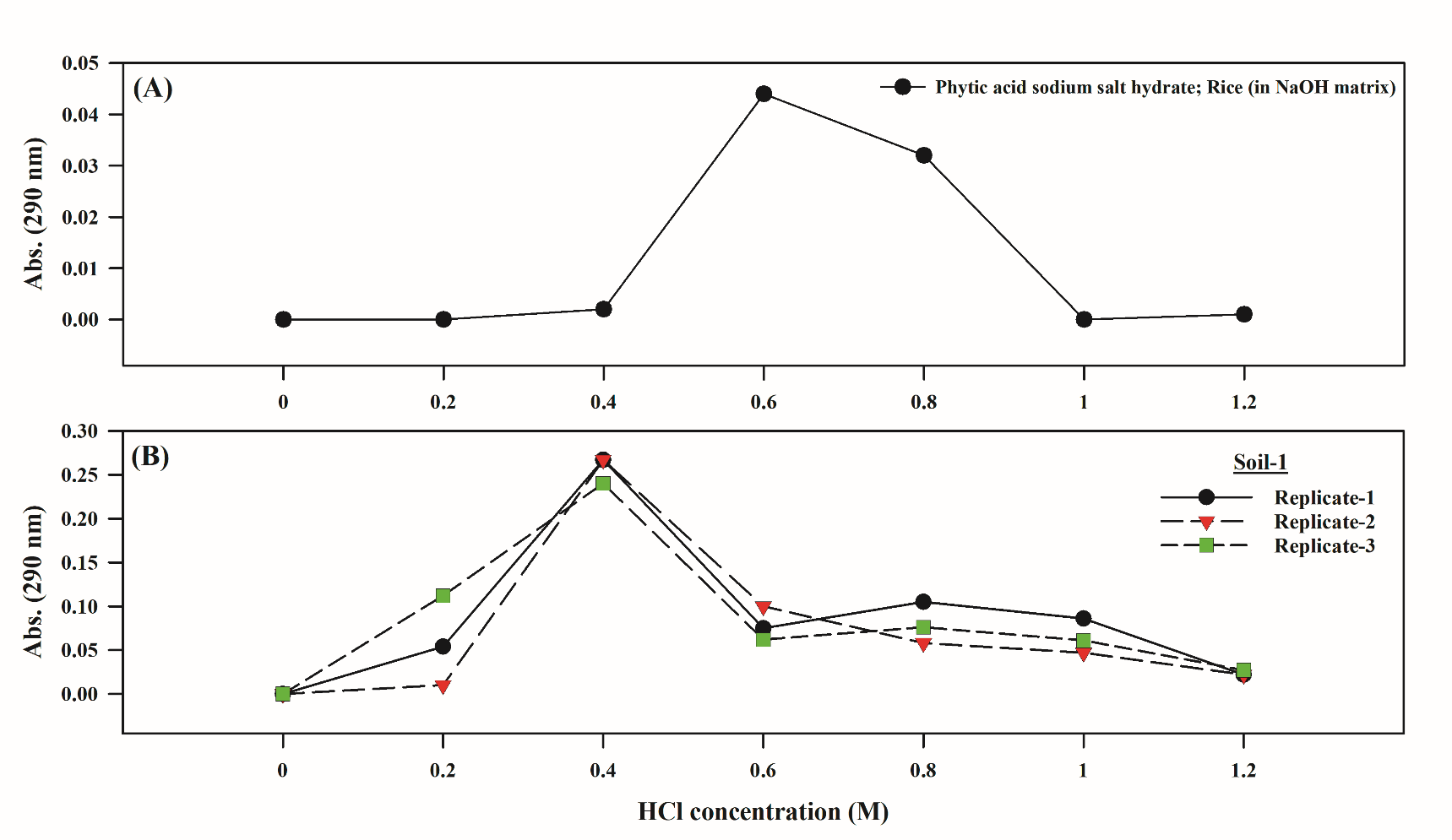


**Figure S1:** Absorbance values (Abs.) for phytate eluted from the ion exchange column with increasing concentrations of HCl from (A) rice and (B) soil sample (Soil-1). The absorbance values for eluates collected from different acid fractions indicate that nearly all of the phytate from the pure compound was eluted with 0.6 M and 0.8 M HCl, whereas in the soil sample, phytate was primarily eluted at the initial phase with 0.2 M and 0.4 M HCl.





**Figure S2:** A calibration curve obtained using the peak area and relative concentration of the pure inositol compound. The equation from the regression analysis was then used to calculate the relative concentration of inositol (derived from phytate) in the samples.





**Figure S3:** The GC-MS chromatogram of extracts of (A & B) soil-1 and (C & D) soil-2. Samples were obtained as eluates from ion exchange chromatography at different HCl concentrations. Each two eluates were pooled as indicated in the figure legend. The eluates were derivatized using the acetic anhydride method by Suzumura & Kamatani. (1993). The chromatograms show the relative abundance of various organic phosphorus compounds (in derivatized form) in non-dephosphorylated (A & C) and dephosphorylated samples (B & D).





**Figure S4:** The GC-MS chromatogram of the derivate of inositol from rice (obtained after dephosphorylation and derivatization of phytate) collected as eluate from ion exchange chromatography at different acid strength (with NaOH as matrix). Eluates collected from different acid fractions were pooled as indicated in the legend. The chromatograms show that almost all of the phytate was eluted with 0.6M and 0.8 M HCl.
